# Treating Early Knee Osteoarthritis with the Atlas® Unicompartmental Knee System in a 26-Year-Old Ex-Professional Basketball Player: A Case Study

**DOI:** 10.1155/2017/5020619

**Published:** 2017-01-26

**Authors:** Konrad Slynarski, Lukasz Lipinski

**Affiliations:** ^1^Lekmed Hospital for Special Surgery, Warsaw, Poland; ^2^Orthopedics and Pediatric Orthopedics Clinic, Medical University of Lodz, Lodz, Poland

## Abstract

Knee osteoarthritis (OA) is a leading cause of disability among adults. Within the affected population, there exists a group of patients who have exhausted conservative treatment options and yet are not ideal candidates for current surgical treatments due to young age, early disease severity, or neutral mechanical knee alignment. For these patients, a new potential treatment option may be considered. We present an interesting case report of a young, ex-professional athlete treated with a minimally invasive load-altering implant (Atlas System) whose young age (26 years), disease status (tibiofemoral kissing lesions), and neutral mechanical limb alignment eliminated all traditional surgical treatment options such as high tibial osteotomy or arthroplasty. At 6 months after surgery, our patient demonstrated positive outcomes improvement in pain, function, and quality of life and had returned to high-impact athletic activity without symptoms. These initial results are promising, and longer follow-up data on the treatment will be necessary.

## 1. Introduction 

Knee osteoarthritis (OA) is a leading cause of disability among adults [1]. Estimated mean age at knee OA diagnosis in the United States is only 53.5 years [2], with a greater number of symptomatic knee OA patients under 65 years of age than over 65 [2, 3] and an annual incidence of knee OA more than five times higher in individuals under 65 years of age than over 65 [2, 3].

The increased prevalence of knee OA in the young population is believed to be due to damage to the articular cartilage caused by repetitive impact and loading [4], biologic changes [5], and altered articular cartilage loading due to joint injuries [6–8]. Prior joint injury such as anterior cruciate ligament rupture or meniscal tear has been shown to accelerate the development of knee OA, with 50% of individuals presenting with the disease just 10 to 20 years following injury [9, 10]. As such joint injuries often occur in the young adult, they can lead to knee OA in individuals as young as 30 or 40 years of age [9].

Treatment options for the young knee OA patient initially consist of nonsurgical conservative modalities, such as activity modification, weight loss, physical therapy, and orthotics, followed by pharmacologic measures such as anti-inflammatories, analgesics, and joint injections. Patients, particularly those with earlier onset OA, often eventually fail conservative treatment [11, 12]. The procedure is considered for younger patients because it can achieve positive mid- to long-term freedom from arthroplasty and may allow a return to high activity levels [13–15]. However, HTO is contraindicated for patients with a neutral axis alignment, and the resultant load transfer may actually accelerate OA progression in the lateral compartment [16].

For these patients, a new potential treatment option may be considered. The recently introduced Atlas System (Moximed, Inc., Hayward, CA, USA) is an implantable, unicompartmental knee joint unloader. Importantly, the device is entirely extracapsular, making the procedure reversible should the patient's disease progress and require future treatment.

We present a novel case report of the Atlas System. The case is unique and intriguing as the young age (26 years) of the patient, disease status (tibiofemoral kissing lesions), and neutral mechanical limb alignment eliminated all traditional surgical treatment options such as HTO or arthroplasty. The patient's status as an ex-professional level athlete and desire to return to high-impact activity add to the case complexity.

## 2. Case Report

### 2.1. Case Presentation

**Figure 1:**
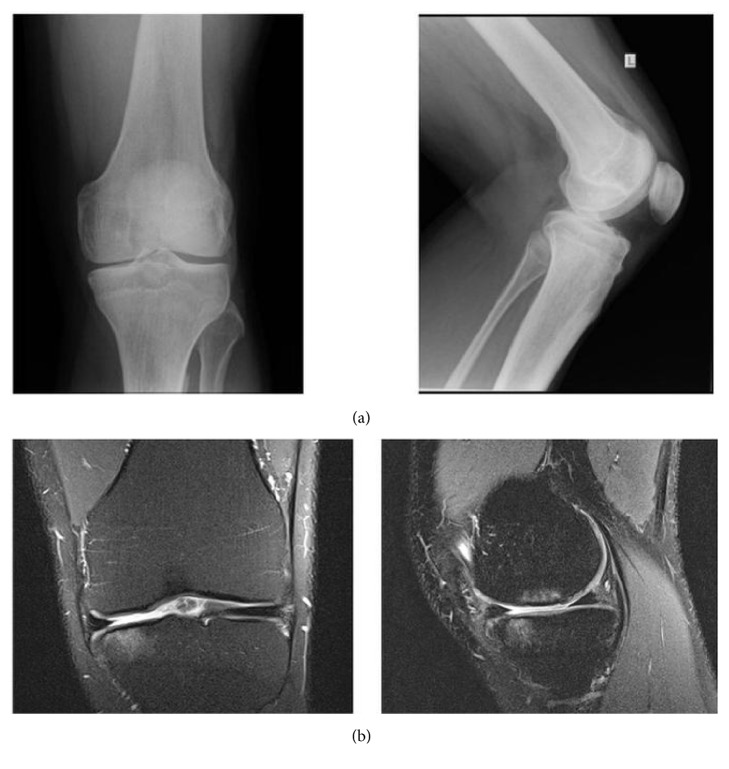
Presurgery anterior and medial radiographs (a) and coronal and sagittal MRIs (b) of the affected left knee.

A 26-year-old male (height: 1.93 m; weight: 95 kg) presented with neutral limb alignment, painful tibiofemoral kissing lesions, and severe knee OA-related activity limitations due to pain in the left knee of one-year duration (Kellgren-Lawrence grade 2) (Figure 1). The knee OA was contained to the medial compartment, and the patient had failed lifestyle/activity modifications, physical therapy, quadriceps strengthening, and analgesics. Preoperative passive range of motion was measured to 140°, and no hyperextension or flexion deformity was recorded. During the orthopedic examination, isolated medial tibiofemoral tenderness was observed. The following symptoms were all absent: patellar tap (no joint effusion), lateral tibiofemoral tenderness, anserine bursa, patellofemoral crepitus, and patellar grind. The ligaments and meniscus were stable. The patient reported mild, continual pain during walking but distance was not limited by the knee pain.

As a former professional league basketball player, the patient indicated a strong desire to return to an active lifestyle including more strenuous activities such as jogging, racquet sports, and basketball, which he was unable to take part in due to pain. After providing written informed consent, he participated in a clinical study that received ethics committee approval and was conducted in compliance with the Ministry of Health and Declaration of Helsinki. The left knee of the patient was treated with the Atlas System, and the patient was followed for a period of six months after surgery.

### 2.2. Device and Surgical Technique

**Figure 2:**
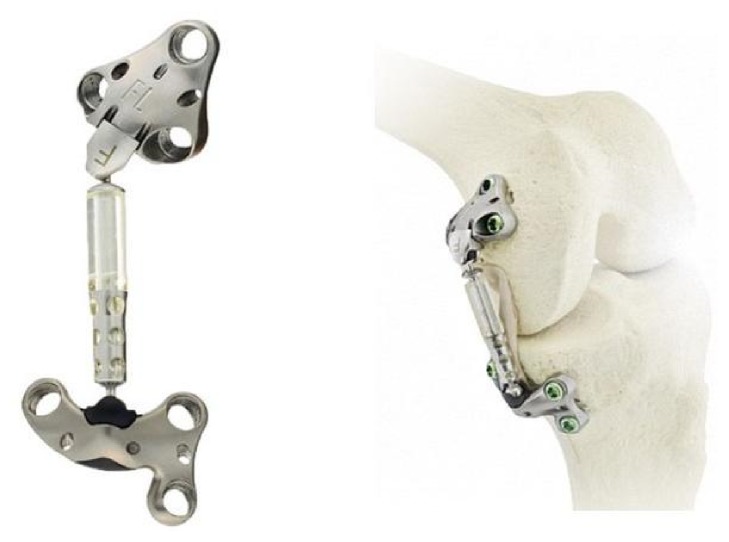
The assembled Atlas Knee System, designed to reduce loading on the affected medial compartment of the knee joint, consists of a load absorber located between femoral and tibial bases.

The Atlas System consists of a cylindrical, polycarbonate urethane (PCU) load absorber located between femoral and tibial bases (Figure 2). The device, located within the subcutaneous tissue on the medial side of the knee, is designed to reduce loading on the affected medial compartment of the knee joint, without transfer of loading to other areas of the joint. The device was inserted through a single incision, guided by direct visualization and palpation of the patient's anatomy. Following identification of the femoral medial epicondyle, adductor magnus tubercle, tibial plateau, joint space, and anterior border of the superficial medial collateral ligament through visualization and palpation, the tibial and femoral fixation points were located, and an absorber length was selected based on the patient's anatomy. A trial device was introduced via two K-wires, and implant function was confirmed through direct visualization checks. Following confirmation of function of the trial device, the final implant was introduced with the femoral base placed deep to the vastus medialis obliquus muscle and the tibial base placed distal of the deep medial collateral ligament and proximal to the insertion of the pes anserine. After installation of the final device, visual confirmation of functional unloading from full extension through deep flexion was performed prior to wound closure. No concomitant intra-articular surgery was performed to ensure that any benefit was due solely to the implant. Postoperatively, the patient was given crutches and told to bear weight as tolerated and to keep the wound protected for an initial 2-week period. Following stitch removal at 2 weeks, the 2-month rehabilitation protocol focused initially on range of motion and daily living activities, followed by muscle strengthening and endurance.

## 3. Results 

**Figure 3:**
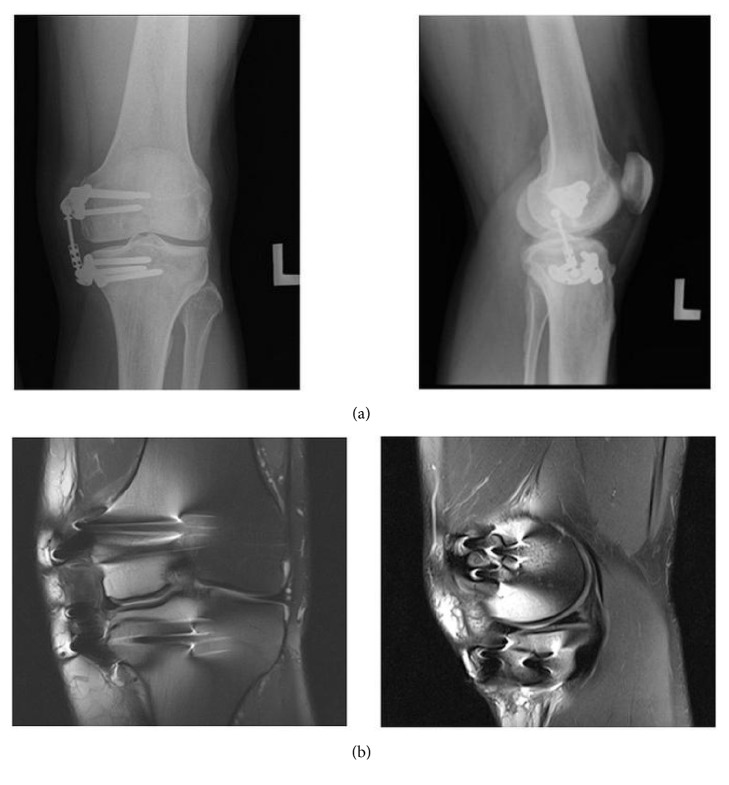
Six-month postsurgery anterior and medial radiographs (a) and coronal and sagittal MRIs (b) of the affected left knee.

The patient experienced no device-related complications during the procedure or in follow-up (Figure 3). Six months following surgery, the patient showed clinically significant improvement (≥10-point improvement) in WOMAC pain and WOMAC function, with final scores of “0” for both domains. Importantly, the patient's KOOS quality of life score had improved by 66.7% (38 to 63). Specifically, the patient's response to the KOOS question, “in general, how much difficulty do you have with your knee?” improved from “extreme” at baseline to “mild” by six months. Physical examination at 6 months revealed full range of motion of 140° of knee flexion. When asked to rate how he was doing, considering all the ways his knee pain affected him, the patient improved from “fair” preoperatively to “very good” postoperatively. The patient's expectations were met: he indicated in an activity and satisfaction survey that he was very satisfied with the results of the procedure, in particular, as he was able to play basketball recreationally and complete his normal daily activities without pain, and would definitely undergo the surgery again for the same condition.

## 4. Discussion 

Osteoarthritis is a common problem afflicting an increasing number of younger, active individuals. The Oxford group in the UK noted that patients with early degenerative changes to their knees should not be ignored, as they can be as symptomatic as those with end-stage disease [17]. The concept of early intervention is increasingly important as some patients with early-to-moderate symptoms of osteoarthritis are unable or unwilling to pursue more advanced surgery, such as HTO, UKA, or TKA. There exists a need for new surgical options that potentially provide symptom relief and early recovery, allow high activity, and maintain all future treatment options.

The Atlas System acts as a shock absorber to unload up to 13 kg of medial compartment joint loading, without transfer of the loading to other healthy areas of the knee joint. This amount of unloading was reported to be similar to that of HTO [18]. Without correction, increased loading on the medial compartment of the knee results in greater disease progression [19, 20] and ultimately the need for joint replacement surgery. As the Atlas System resides in the subcutaneous tissues outside of the joint capsule there is no breech of joint capsular space, nor is bone resection required, thus creating a reversible procedure and maintaining all future treatment options.

As this was a novel, early experience with the device, the rehabilitation protocol was not well-studied previously. The patient was allowed to bear full weight immediately (as tolerated) after surgery. He was discharged with crutches as a reminder to limit early activity and encourage full wound healing. Bracing was not employed after surgery, and early passive range of motion was recommended. The recovery protocol was positive, as the patient demonstrated clinically meaningful outcomes improvement and had returned to high-impact recreational sports (basketball, jogging) by six months after surgery.

Recently, authors presented improved clinical outcomes [21] in a 40-patient series of the Atlas System, with WOMAC pain and function scores improving from 52 ± 12 and 52 ± 17, respectively, at baseline to 15 ± 15 and 19 ± 17 at six months. Knee Society pain and function scores also improved, from 62 ± 15 and 71 ± 18 at baseline to 91 ± 12 and 98 ± 4 at six months. Additionally, the unicompartmental Atlas System was used in patients with cartilage defects or degenerative meniscus [22].

The results of this case study, demonstrating positive outcomes improvement in pain, function, quality of life, and activity level at an initial 6-month time period, indicate promising results in a highly unique case of a young, ex-professional athlete with early knee OA.

## References

[1] Guccione A. A., Felson D. T., Anderson J. J. (1994). The effects of specific medical conditions on the functional limitations of elders in the Framingham study. *American Journal of Public Health*.

[2] Losina E., Weinstein A. M., Reichmann W. M. (2013). Lifetime risk and age at diagnosis of symptomatic knee osteoarthritis in the US. *Arthritis Care & Research*.

[3] Howden L. M., Meyer J. A. (2011). *Age and Sex Composition: 2010*.

[4] Buckwalter J. A., Lane N. E. (1997). Athletics and osteoarthritis. *American Journal of Sports Medicine*.

[5] Kramer W. C., Hendricks K. J., Wang J. (2011). Pathogenetic mechanisms of posttraumatic osteoarthritis: opportunities for early intervention. *International Journal of Clinical and Experimental Medicine*.

[6] Andriacchi T. P., Koo S., Scanlan S. F. (2009). Gait mechanics influence healthy cartilage morphology and osteoarthritis of the knee. *The Journal of Bone & Joint Surgery—American Volume*.

[7] Cattano N. M., Barbe M. F., Massicotte V. S. (2013). Joint trauma initiates knee osteoarthritis through biochemical and biomechanical processes and interactions. *OA Musculoskeletal Medicine*.

[8] Roos E. M. (2005). Joint injury causes knee osteoarthritis in young adults. *Current Opinion in Rheumatology*.

[9] Lohmander L. S., Östenberg A., Englund M., Roos H. (2004). High prevalence of knee osteoarthritis, pain, and functional limitations in female soccer players twelve years after anterior cruciate ligament injury. *Arthritis and Rheumatism*.

[10] Lohmander L. S., Englund P. M., Dahl L. L., Roos E. M. (2007). The long-term consequence of anterior cruciate ligament and meniscus injuries: osteoarthritis. *American Journal of Sports Medicine*.

[11] Hochberg M. C., Altman R. D., Brandt K. D. (1995). Guidelines for the medical management of osteoarthritis. Part II. osteoarthritis of the knee. *Arthritis & Rheumatism*.

[12] Hunter D. J., Felson D. T. (2006). Osteoarthritis. *British Medical Journal*.

[13] Bode G., von Heyden J., Pestka J. (2015). Prospective 5-year survival rate data following open-wedge valgus high tibial osteotomy. *Knee Surgery, Sports Traumatology, Arthroscopy*.

[14] Saito T., Kumagai K., Akamatsu Y., Kobayashi H., Kusayama Y. (2014). Five- to ten-year outcome following medial opening-wedge high tibial osteotomy with rigid plate fixation in combination with an artificial bone substitute. *The Bone & Joint Journal*.

[15] Salzmann G. M., Ahrens P., Naal F. D. (2009). Sporting activity after high tibial osteotomy for the treatment of medial compartment knee osteoarthritis. *The American Journal of Sports Medicine*.

[16] Duivenvoorden T., Brouwer R. W., Baan A. (2014). Comparison of closing-wedge and opening-wedge high tibial osteotomy for medial compartment osteoarthritis of the knee: a randomized controlled trial with a six-year follow-up. *The Journal of Bone & Joint Surgery—American Volume*.

[17] Jones L. D., Bottomley N., Harris K., Jackson W., Price A. J., Beard D. J. (2016). The clinical symptom profile of early radiographic knee arthritis: a pain and function comparison with advanced disease. *Knee Surgery, Sports Traumatology, Arthroscopy*.

[18] Becher C., Huelsmann J., Ettinger M., Fleischer B., Niemeyer P., Bode G. (2016). Comparing established and emerging surgical options for load reduction of the medial knee: a biomechanical study. *Knee Surgery, Sports Traumatology, Arthroscopy*.

[19] Chehab E. F., Favre J., Erhart-Hledik J. C., Andriacchi T. P. (2014). Baseline knee adduction and flexion moments during walking are both associated with 5 year cartilage changes in patients with medial knee osteoarthritis. *Osteoarthritis and Cartilage*.

[20] Miyazaki T., Wada M., Kawahara H., Sato M., Baba H., Shimada S. (2002). Dynamic load at baseline can predict radiographic disease progression in medial compartment knee osteoarthritis. *Annals of the Rheumatic Diseases*.

[21] van der Merwe W., Slynarski K., Walawski J., Smigielski R. (2016). Preliminary results: 40 patient, multi-center, prospective study of an implantable, extra-capsular, polycarbonate urethane knee unloader. *Knee Surgery, Sports Traumatology, Arthroscopy*.

[22] Slynarski K., Lipinski L., Krzesniak A. (2016). Feasibility of joint unloading for degenerative medial meniscus, cartilage defects, and OA. *Knee Surgery, Sports Traumatology, Arthroscopy*.

